# The Burden of Cholera in Uganda

**DOI:** 10.1371/journal.pntd.0002545

**Published:** 2013-12-05

**Authors:** Godfrey Bwire, Mugagga Malimbo, Brian Maskery, Young Eun Kim, Vittal Mogasale, Ann Levin

**Affiliations:** 1 Control of Diarrheal Diseases Section, Ministry of Health, Kampala, Uganda; 2 Epidemiological Surveillance Division, Ministry of Health, Kampala, Uganda; 3 International Vaccine Institute, Seoul, Korea; 4 Independent Consultant, Bethesda, Maryland, United States of America; Massachusetts General Hospital, United States of America

## Abstract

**Introduction:**

In 2010, the World Health Organization released a new cholera vaccine position paper, which recommended the use of cholera vaccines in high-risk endemic areas. However, there is a paucity of data on the burden of cholera in endemic countries. This article reviewed available cholera surveillance data from Uganda and assessed the sufficiency of these data to inform country-specific strategies for cholera vaccination.

**Methods:**

The Uganda Ministry of Health conducts cholera surveillance to guide cholera outbreak control activities. This includes reporting the number of cases based on a standardized clinical definition plus systematic laboratory testing of stool samples from suspected cases at the outset and conclusion of outbreaks. This retrospective study analyzes available data by district and by age to estimate incidence rates. Since surveillance activities focus on more severe hospitalized cases and deaths, a sensitivity analysis was conducted to estimate the number of non-severe cases and unrecognized deaths that may not have been captured.

**Results:**

Cholera affected all ages, but the geographic distribution of the disease was very heterogeneous in Uganda. We estimated that an average of about 11,000 cholera cases occurred in Uganda each year, which led to approximately 61–182 deaths. The majority of these cases (81%) occurred in a relatively small number of districts comprising just 24% of Uganda's total population. These districts included rural areas bordering the Democratic Republic of Congo, South Sudan, and Kenya as well as the slums of Kampala city. When outbreaks occurred, the average duration was about 15 weeks with a range of 4–44 weeks.

**Discussion:**

There is a clear subdivision between high-risk and low-risk districts in Uganda. Vaccination efforts should be focused on the high-risk population. However, enhanced or sentinel surveillance activities should be undertaken to better quantify the endemic disease burden and high-risk populations prior to introducing the vaccine.

## Author Summary

Uganda has reported cholera cases to the World Health Organization every year since 1997. Thus, the country may consider the introduction of a WHO-prequalified oral cholera vaccine. This article reviews cholera surveillance data from 1997–2010 with a focus on the 2005–2010 time period to identify high risk populations that may be targeted for preventive vaccination campaigns. We estimated that an average of about 61–182 deaths occur each year. Most cases (81%) occurred in a relatively small number of districts comprising just 24% of Uganda's total population. While there is a clear distinction between low and high-risk districts, sentinel surveillance would help to better quantify the burden in endemic districts. An economic analysis should also be undertaken prior to making a decision to introduce a cholera vaccine.

## Introduction

**Figure 1:**
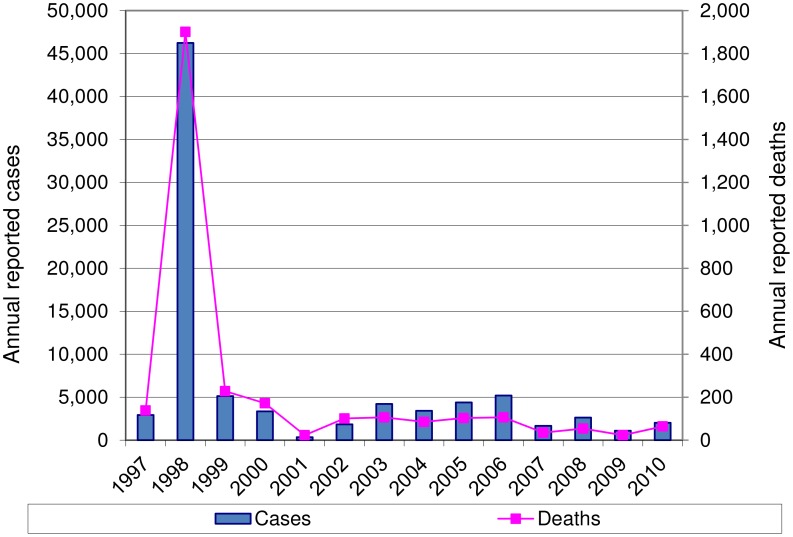
Annual number of cholera cases and deaths reported in Uganda 1997–2010. There was a major outbreak in 1998 and a fluctuating, but persistent burden of cholera in the years since the outbreak.

Cholera was first reported in Uganda in 1971, when 757 cases were reported to the World Health Organization (WHO). During the subsequent years up to 1993, Uganda reported cholera cases every 2–4 years to the WHO. From 1994 to 1998, cholera was reported annually in Uganda [1]. In 1998, Uganda reported almost 50,000 cases with incidence throughout the country [2]. The reported incidence has fluctuated between 250 and 5,000 cases every year since 2000 (Figure 1). The reported case fatality ratio has decreased from 4–7% in the late 1990s to about 2–3% during 2004–2010.

Cholera in Uganda appears to be largely an epidemic disease. However, endemic cholera occurs in high-risk areas along the southwestern border with DRC and in Kampala city slums. Endemic cholera is commonly noted before and during the rainy season, from December through March. Epidemic cholera can occur any time, but is often associated with extreme rain events or water supply disruptions.

The frequency of reported cholera cases varies among districts in Uganda. The highest risk areas include the border areas with the Democratic Republic of Congo (DRC), Sudan, and Kenya as well as urban slums in Kampala. Displaced populations and their neighboring communities are at elevated risk. The ongoing migration of people into and within Uganda can lead to rapid spread of the disease. The African Great Lakes, including Lake Albert and Lake Victoria on the border of Uganda, may provide a reservoir for cholera bacteria. Further, increases in incidence among the nations bordering these lakes have been shown to be correlated with El Niño warm weather events [3]. The WHO [4] has recently revised its guidelines and states in a position paper that cholera vaccines should be used in combination with other prevention and control strategies in areas where the disease is endemic. “Endemic” is defined as areas with occurrence of culture-confirmed cholera in at least three of the previous five years. The WHO also recommends that cholera vaccines should be considered for preemptive use in areas at risk for epidemic cholera as long as vaccination does not interfere with efforts to treat cholera cases, improve water and sanitation, and mobilize communities. The vaccine may also be considered for reactive use if local infrastructure is sufficient to conduct mass campaigns depending on the current and historical epidemiology.

There is a dearth of information about the burden of cholera in low-income countries such as Uganda. A more accurate picture of this burden is particularly important because it can be used to inform cholera prevention and control intervention questions: whether or not to introduce vaccination as a complement to other cholera prevention and control interventions, where and when it would be most effective to do so, and what demographic population should be targeted. This article presents available disease burden data for Uganda that may help inform such questions.

## Methods

### Study design

This is a retrospective study in which we collected data from Uganda's health information management system and Diarrheal Disease Control program. District-specific data were used to classify districts as endemic or non-endemic based on the WHO criterion and to identify high-incidence districts. A convenience sample of more detailed data from individual cholera outbreaks were summarized to estimate the age distribution of reported cholera cases and to develop epidemic curves. Because the Ugandan surveillance system is designed primarily to identify and respond to cholera outbreaks, a sensitivity analysis was performed to explore the potential limitations of the existing surveillance system to identify cases outside of recognized outbreaks.

### Current surveillance practices in Uganda

These data were used to compile national statistics and for reporting to the World Health Organization's (WHO) Weekly Epidemiological Record [5]. The cholera case definition was based on WHO criteria, depending on whether or not cholera is endemic in the area:

- In non-endemic areas: “a patient aged 5 years or more develops severe dehydration or dies from acute watery diarrhea.”
- In endemic areas: “a patient aged 2 years or more develops severe dehydration or dies from acute watery diarrhea.” [6]

The identification of such cases should have triggered laboratory investigation. A cholera outbreak was confirmed when *Vibrio cholerae* O1 or O139 was isolated from at least one stool sample. Only cases meeting the standard case definition above were investigated and included in the official cholera data.

### Laboratory methods

Summary laboratory data were obtained from the Head of the Central Public Health Laboratory from the Ministry of Health. Prior to analysis, stool samples from suspected cholera patients were transported from the field in Cary Blair transport media. Culture plates were set at 37°C overnight (for 18–24 hours) using three culture media: TCB, XLD and MacConkey. Biochemical identification of cholera organisms were based on Oxidase or Indole tests. Polyvalent antisera were used to differentiate between the Inaba and Ogawa serotypes, and specific monovalent tests further confirmed which of the Inaba, Ogawa or O139 (Bengal) strains caused disease. Isolates were refrigerated at −80°C and sent to the WHO's collaborating laboratory (Unité de La Rage, Institut Pasteur, Paris, France) for quality control.

### Data collection

District-specific data were abstracted from the Uganda Ministry of Health, Health Management Information System disease surveillance database for the period 2005–2010. The 2005–2010 period was chosen based on the WHO criteria [4] for identifying endemic cholera (i.e. areas in which cholera has been reported in three of the previous five years.) The districts shown are based on the 2002 district boundaries, which were in existence during the most recent census. Cases in new districts created after 2005 were apportioned back to the 2002 districts.

Age-specific morbidity and mortality data are stored at the district level. These ‘line list’ records include: patient age, outcome of treatment (i.e. discharge or death), and date of admission or death (for suspected cholera patients who die prior to seeking treatment). We were able to obtain these data from 15 outbreaks, which occurred in 12 districts spanning the time period from 2002–2010. In total, the line list data included records of 6,125 cholera cases with at least 154 deaths. The actual number of deaths could not be ascertained because some of the records lack data on patient outcomes. These records included seven instances in which death occurred in the community, (i.e. prior to receiving treatment). In addition, there were 923 records with data on inpatient and outpatient treatment and the duration of inpatient treatment.

For this retrospective analysis, the study team compiled data from samples that were previously collected and analyzed as part of routine surveillance activities.

### Data analysis

The incidence of hospitalized cholera was estimated by district based on the annual average number of cases reported over the six-year period from 2005–2010. The district-specific reporting does not include data by age group. Thus, the age distribution of cases was estimated based on the 15 line lists. It was assumed that these 15 outbreaks were representative of the age distribution of cholera incidence in Uganda. The numbers of cases by age group were calculated from the product of total cases and the national average percentage distribution of cases by age from the line list data. Age-specific incidence rates were then calculated by dividing the age-specific cases by the age-specific populations (2010 UN population data). All analyses and graphs were produced with Microsoft Excel (Microsoft, Redmond, WA) and maps were created with ArcGIS (ESRI, Redlands, CA). Cholera case fatality rates were estimated from the number of reported cases and deaths by age group from the line list data. The Fisher's exact test was used to compare case fatality rates across age groups. Statistical analyses were performed using STATA software (Version 8, College Station, TX, USA).

### Sensitivity analysis

In addition to hospitalized cases, we also estimated the number of non-hospitalized or non-reported cholera cases in Uganda. In a recent analysis, Kirigia et al. [7] estimated that 10% of cholera cases could be classified as severe and require hospitalization. In addition, Poulos et al. [8] reported that 22–38% of cholera patients were hospitalized during multi-site surveillance studies conducted in Jakarta, Indonesia and Kolkata, India. In Uganda, patients with mild diarrhea often do not seek formal seek care, but do receive oral rehydration therapy at home. In this analysis, we assumed that the official statistics include 25% of cholera patients with severe cholera who would seek treatment and be reported in official statistics and that 75% took oral rehydration therapy at home. This assumption is greater than that assumed by Kirigia et al., but falls at the lower end of the actual data presented by Poulos et al. Thus, we estimated that there were three non-hospitalized cholera cases requiring treatment at home per one officially reported case. Estimation of asymptomatic cholera infections were omitted from this analysis.

Since the reported numbers of deaths were based on individually-identified cholera patients, these reports should be a lower bound. While deaths that occurred outside treatment facilities were included in official reports when identified, it remains likely that some cholera deaths were missed and not reported in official statistics. In a recent study in neighboring Kenya, an active case finding exercise identified a 200% increase in the number of cholera deaths that occurred during a 2008 cholera outbreak [9]. This is a worst-case-scenario, since the outbreak occurred during a chaotic period of post-election violence. However, in addition to deaths that were missed during outbreaks, isolated cholera deaths that occur outside of recognized outbreaks may also contribute to underreporting in official statistics. As an upper bound estimate of the annual number of cholera deaths in Uganda, we applied the 200% correction factor from the Kenya study to the number of cholera deaths identified in Uganda. In addition, for an upper bound estimate of the number of hospitalized cases, we assumed that a number of severe acute watery diarrheal cases that occurred outside of recognized outbreaks were the result of infection by *Vibrio cholerae*. These endemic cholera cases have frequently been omitted from totals in other cholera endemic countries [10], [11]. Thus, we assumed the number of reported hospitalized cases may have only been about 50% of the actual cases although this rate is difficult to estimate in the absence of sentinel surveillance data.

## Results

The Ministry used laboratory confirmation for a sub-sample of suspected cholera cases. In 2008, the national laboratory confirmed that 71 cases out of 150 tested samples were due to *V. cholerae* (42%). The relatively low level of confirmed cases could be due to poor specimen collection, transportation or the less specific standard case definition, which tends to include severe forms of other acute watery diarrhea. Importantly, the laboratory also ruled out cholera as the causative organism for at least three diarrheal disease outbreaks [personal communication: October 31, 2011, Mr. Ateki Kagirita, Head Central Public Health Laboratory]. These findings are comparable to those reported during the 2002–03 cholera outbreaks in Uganda in which researchers found that 52% of the suspected cholera patients had positive stool samples [12]. During 2008–09, about 82% of the confirmed cholera cases were attributed to the Ogawa serotype and the remaining 18% to the Inaba serotype.

**Figure 2:**
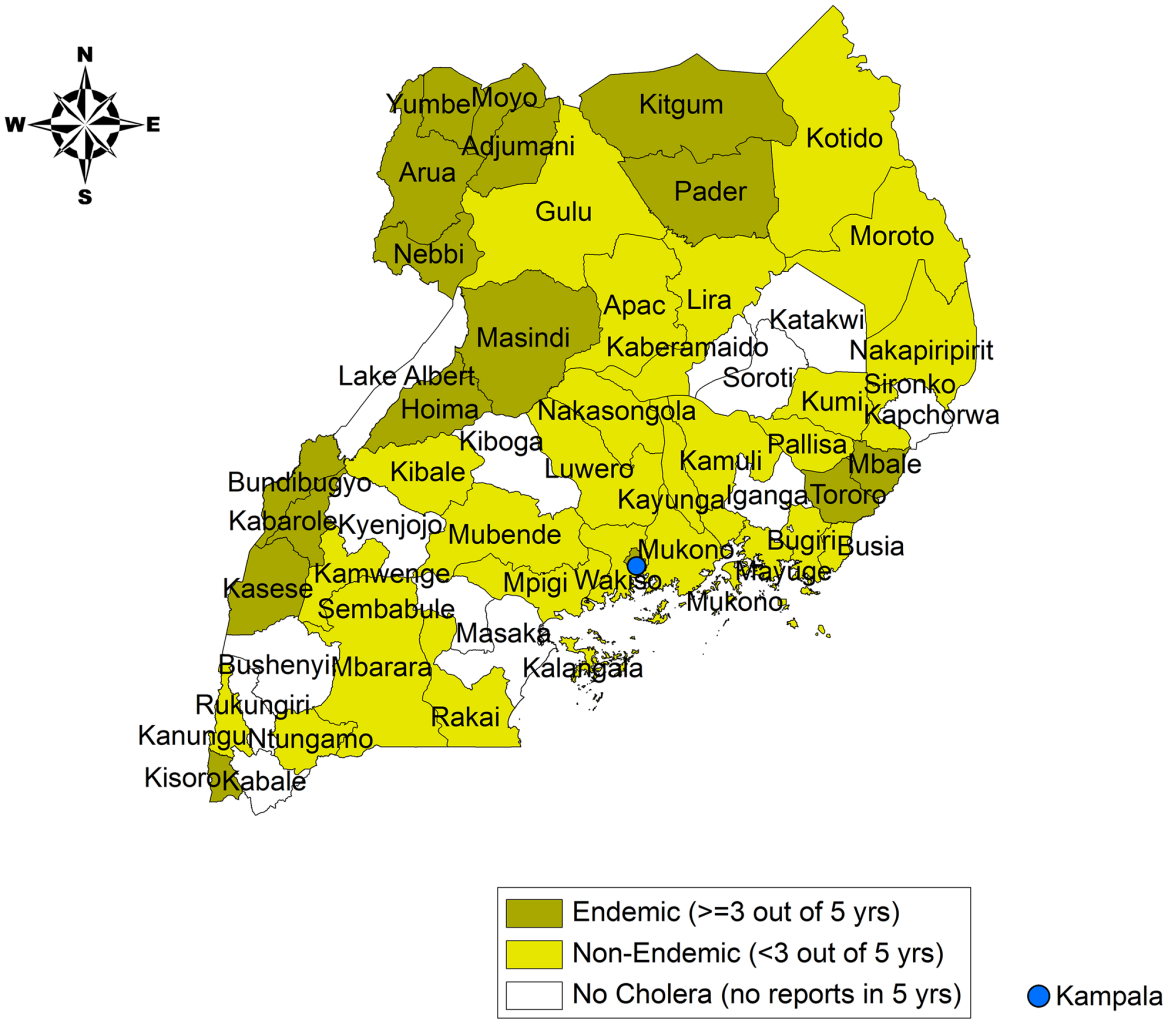
Districts with reported cholera cases from 2005 to 2010 (endemic and non-endemic districts). Some districts may be considered endemic for cholera based on a history of cholera incidence during three of the five years from 2005–2010. Other districts have demonstrated cholera on a less consistent basis, while a few districts did not report any cases between 2005–2010.

There were relatively few districts within Uganda in which cholera was confirmed every year. Kasese district, on the border with DRC, and the Kampala slums both reported cholera during at least five of the six years between 2005–2010. *Figure 2* identifies districts that met the WHO definition of endemicity for cholera, i.e. where cholera was confirmed in three of the previous five years. Districts in which cholera was reported, but in fewer than three of the previous five years, were identified as non-endemic. The remaining districts did not report cholera from 2005 through 2010.

**Figure 3:**
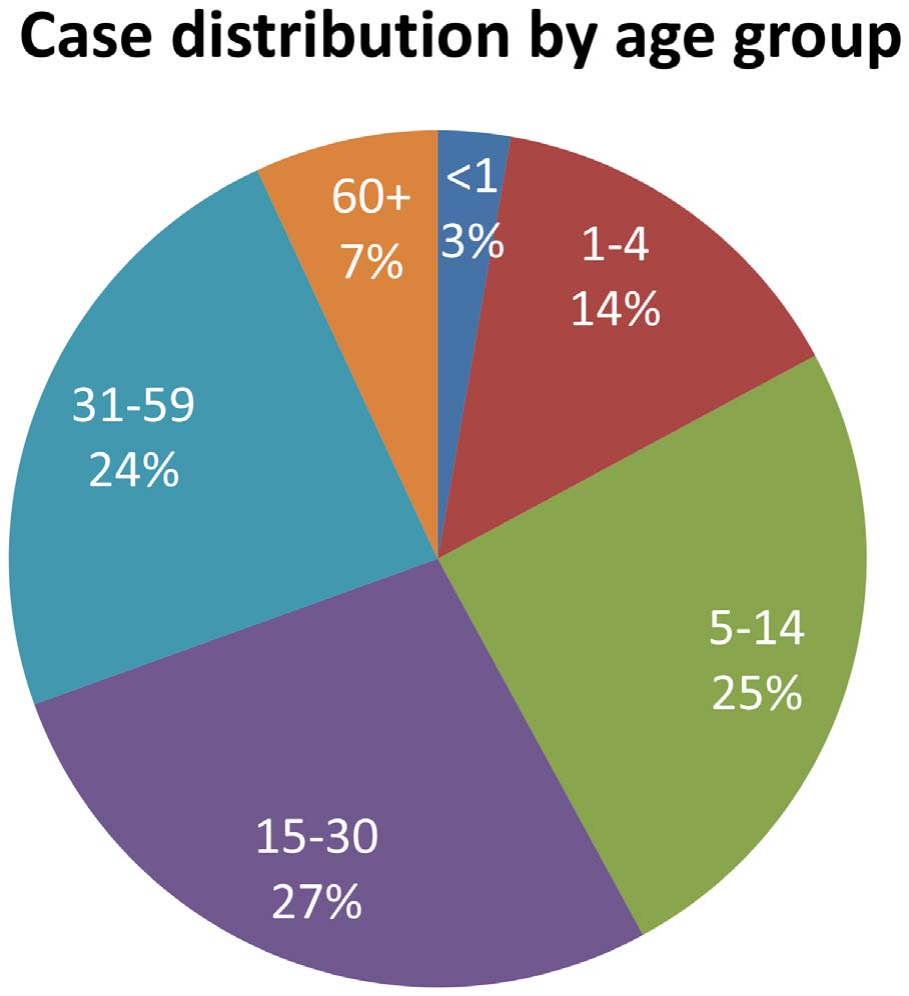
Age breakdown of cholera cases from non-random sample of cholera outbreaks, 2002–2010. The percentage distribution of cholera cases in Uganda is similar to the overall age distribution of the population.

In addition to the slums of Kampala, the cholera-outbreak-prone districts were mostly located along the western and northern borders. In addition to cholera, these districts have also been prone to outbreaks of other waterborne diseases such as typhoid, shigellosis, and hepatitis E. Cholera affected all age groups in Uganda. Based on existing surveillance data, the age distribution of cholera followed the age distribution of the population (*Figure 3*). The gender distribution among cholera cases in Uganda was 54% female, 46% male.

**Figure 4:**
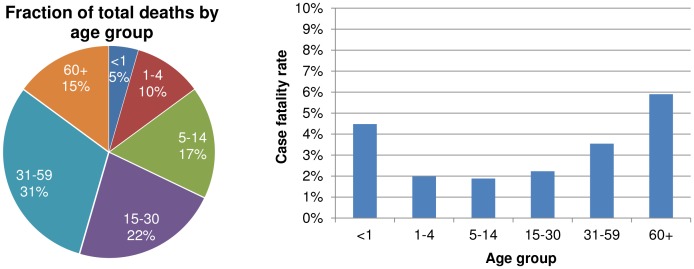
Distribution of deaths by age group and average case fatality rate by age group. The age distribution of cholera deaths varies slightly from age distribution of cases because the case fatality rates are higher infants and the elderly relative to other age groups.

The distribution of cholera deaths by age group and the average case fatality rates during cholera outbreaks are summarized in *Figure 4*. The case fatality rate was greater for elderly persons (p<0.001) than other groups. However, mortality occurred in all age groups.

The breakdown for inpatient versus outpatient treatment and length of hospitalization was not typically available in the line list data. Among the 923 records with such data, the hospitalization rate was about 90% and average duration of inpatient treatment was 2.4 days.

**Figure 5:**
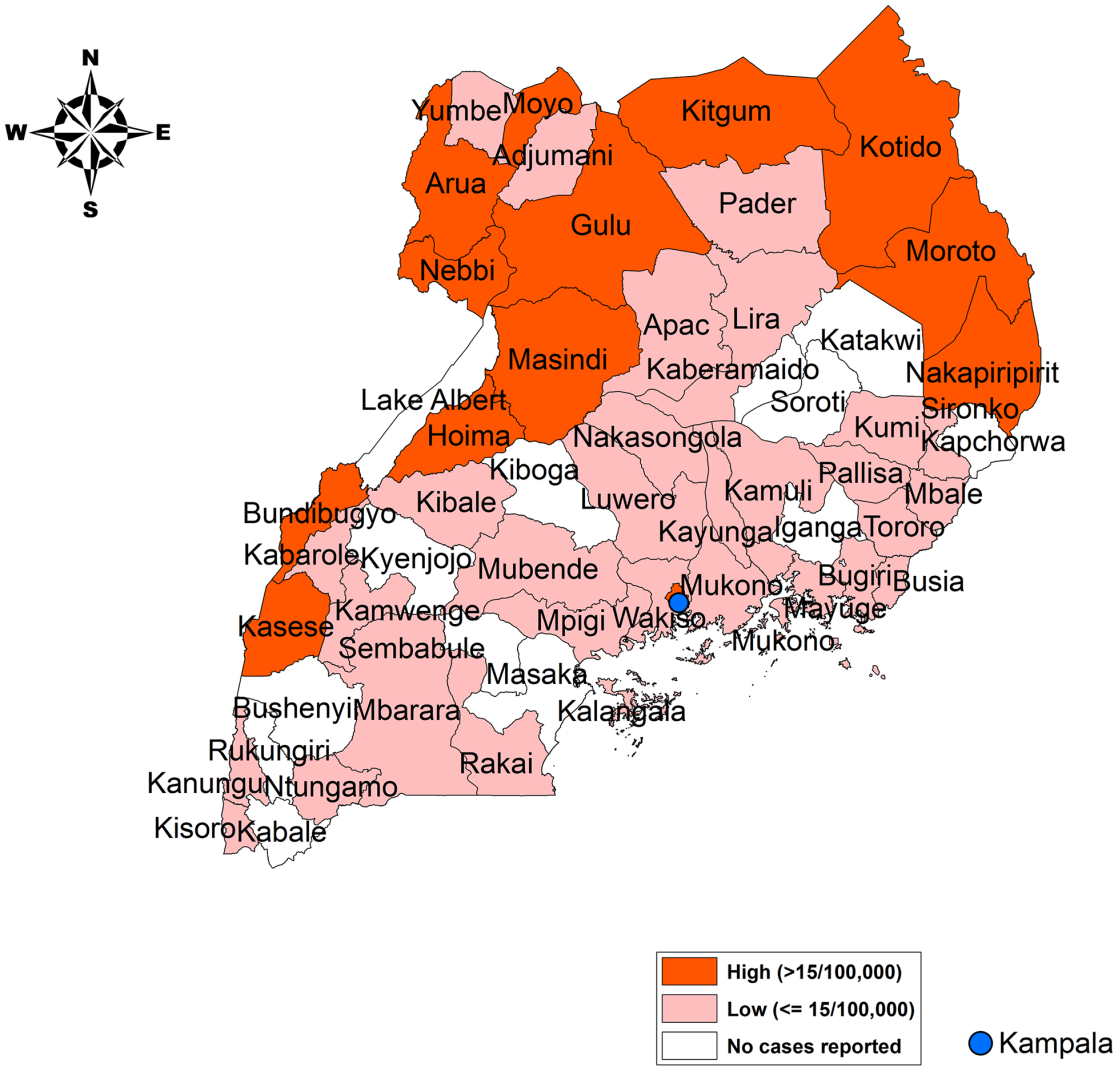
Map of reported cholera incidence by district, hospitalized cases, 2005–10. The estimated incidence by district varies considerably in Uganda with a distinct pattern of low-incidence versus high incidence districts.

An annual average incidence rate of cholera was estimated for each district based on the average number of cases reported during 2005–2010. In *Figure 5*, the districts are subdivided into three categories, a high incidence category in which the average annual reported incidence was greater than 15 cases per 100,000 persons, a low incidence category in which the average annual incidence was greater than zero but less than 15 cases per 100,000 persons, and, finally, a category in which no cases were reported during the previous six years.

Based on this threshold, 13 of the 56 districts that existed in 2005 were at high risk for cholera. These high-risk districts included an estimated 2010 population of 7.6 million people (24% of the population). Another 31 districts (18.0 million people, 57% of the population) were considered at “low risk” and 12 districts (6.1 million, 19% of the population) did not report any cases in the past six years (*Table 1*). Thus, the majority of Uganda's population (76%) resides in districts that can be considered at “low-risk” for cholera.

**Table 1:** Estimated annual cholera incidence rates, number of cases and death by district risk category and age group.

District risk category	Age group	Population	Reported cases	Total annual cases (range)	Estimated incidence per 1,000 (range)	Annual deaths (reported)	Annual deaths (range)
High	<1	369,000	50	50–200	14–55	1	1–4
	1–4	1,208,000	363	360–1,400	30–120	1	1–4
	5–14	2,386,000	538	540–2,200	23–90	8	8–24
	15+	4,172,000	1,285	1,300–5,100	31–120	38	38–115
	All ages	8,135,000	2,235	2,200–8,900	28–110	49	49–148
Low or Unknown	<1	1,164,761	12	12–48	0.2–0.8	0.23	0–1
	1–4	3,809,393	85	85–340	1.5–6	2	2–5
	5–14	7,527,670	126	130–550	2–8	3	3–8
	15+	13,160,134	301	300–1,200	5–20	7	7–20
	All	25,661,958	524	520–2,100	8.8–35	11	11–34
All districts	<1	1,534,000	62	62–250	1–4	1	1–4
	1–4	5,017,000	448	450–1,800	7.5–30	3	3–10
	5–14	9,914,000	664	660–2,700	11–44	11	11–33
	15+	17,332,000	1,586	1,600–6,300	26–105	45	45–135
	All	33,797,000	2,760	2,800–11,000	46–180	61	61–182

When considering district-specific incidence rates, it is important to note that cholera risk varied even within districts. During the 2010 cholera outbreak in the northeastern districts of Moroto and Kotido, more than 85% of cases in each district occurred in fewer than 27–33% of the sub-counties [13].

### Estimated annual numbers of cases and deaths

The estimated annual number of cholera cases by age and by district risk group is shown in *Table 1*. Inclusive of unreported cases treated at home, our estimated annual average number of cases was around 11,000, with around 81% of the cases occurring in the high risk districts.

On average, about 61 cholera deaths were reported per year during 2005–2010. Using the 200% correction factor reported from the Kenyan study [9], the potential range of annual cholera mortality is 61–182 deaths per year.

### Epidemic cholera

**Figure 6:**
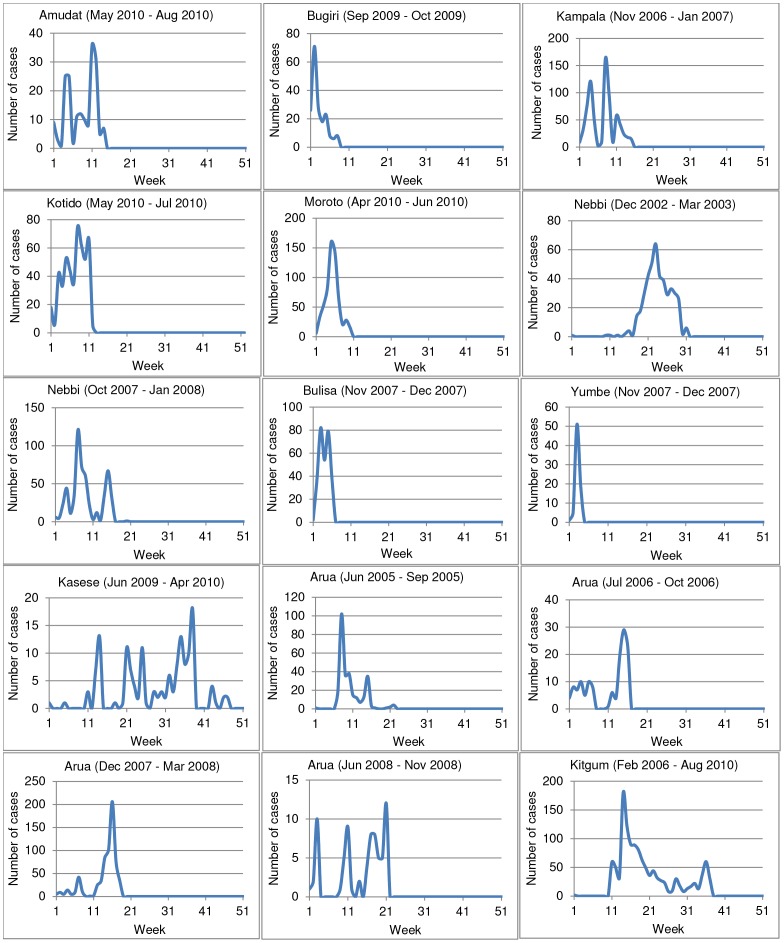
Epidemic curves of cholera outbreaks in Uganda, 2002–2010. The epidemic curves from a convenience sample of fifteen outbreaks shows both large and small outbreak have occurred during the period 2002–2010. There is considerable variation in the duration of outbreaks with longer outbreaks in districts with higher average annual incidence rates.

The epidemic curves for fifteen cholera outbreaks that occurred in Uganda between 2002 and 2010 are shown in *Figure 6*. The average duration of the 15 outbreaks was about 15 weeks from the identification of the first case through the identification of the last case. The range of outbreak duration was between 4 weeks and 44 weeks (*Table 2*). Almost half of the observed cases (43%) occurred within six weeks of the first case. It should be noted that the cases reported for Kasese were more likely to be representative of endemic disease, as this district is one of the few that report cases on an ongoing basis. Arua district reported four outbreaks between 2005 and 2008, but weekly cases declined from the peak observed in early 2008.

**Table 2:** A non-random sample of outbreaks in Uganda, 2002–2010.

District	Time period (month)	Time period (week)	Cases	District	Time period (month)	Time period (week)	Cases
Amudat	May 2010–Aug 2010	14 weeks	185	Kasese	Jun 2009–Apr 2010	44 weeks	148
Arua	Jun 2005–Sep 2005	16 weeks	289	Kitgum	Feb 2006–Oct 2006	37 weeks	1,245
Arua	Jul 2006–Oct 2006	16 weeks	135	Kotido	May 2010–Jul 2010	12 weeks	491
Arua	Dec 2007–Mar 2008	18 weeks	663	Moroto	Apr 2010–Jun 2010	10 weeks	610
Arua	Jun 2008–Nov 2008	21 weeks	73	Nebbi	Dec 2002–Mar 2003	17 weeks	437
Bugiri	Sep 2009–Oct 2009	8 weeks	188	Nebbi	Oct 2007–Jan 2008	16 weeks	551
Bulisa	Nov 2007–Dec 2007	6 weeks	291	Yumbe	Nov 2007–Dec 2007	4 weeks	75
Kampala	Nov 2006–Feb 2007	15 weeks	726				

## Discussion

This retrospective analysis showed that there is a clear subdivision between high-risk districts and low-risk districts in Uganda with about 24% of the population residing in high-risk districts accounting for 81% of the average reported cholera burden. These high-risk districts may be considered for preventive cholera vaccination campaigns in combination with other cholera control activities.

Cholera affects all age groups in Uganda. The age distribution of cases matched the population distribution. This may be due to low levels of background immunity, so that the entire population is equally susceptible. This age distribution deviates from age distributions in other cholera-endemic areas, where young children tended to be at greater risk when systematic surveillance was conducted [14]. Systematic sampling of diarrheal cases from endemic areas has never been attempted in Uganda and may reveal that outbreak-based surveillance findings are not representative of the true cholera burden. A comparison of age-specific cholera incidence rates from Bangladesh demonstrated that the average age of cholera infection was much higher during outbreaks [15] than for endemic cholera [16].

Outbreak data from the sub-district level suggests that there may be considerable heterogeneity of cholera incidence. Thus, surveillance efforts and reporting should be improved to facilitate better epidemiological characterization of cholera incidence and improved targeting of interventions to reach those at greatest risk.

The estimate of 61 deaths per year involves accreditation of all cholera deaths to specific individuals, either at treatment centers or in the community. This is a relatively small fraction of the estimated 30,000 diarrheal deaths per year in Uganda (exclusive of deaths attributed to cholera and bloody diarrhea) [17]. It is certainly possible that a significant proportion of these 30,000 deaths was caused by unrecognized cholera than would be estimated from individually identified deaths. Although the outbreak response focus of cholera surveillance in Uganda may be insufficient to accurately estimate the numbers of cases and deaths caused by cholera, these data are very useful for identifying areas to target for surveillance in consideration of future vaccine introduction.

In order to better quantify the burden of cholera in Uganda, sentinel site surveillance should be undertaken in at least two regions with districts at high risk for cholera for a period of at least two years. It would be better to continue surveillance for at least five years, since cholera incidence is highly variable from year to year. The Ministry of Health is participating in the AFRICHOL cholera surveillance in Africa project (www.africhol.org), led by Agence de Médecine Préventive (AMP) and the African Field Epidemiological Network (AFENET), which is an African-based non-government organization working to improve field epidemiology and public health laboratories in sub-Saharan Africa. As part of this project, enhanced cholera surveillance is being conducted in five districts in Eastern Uganda (Mbale, Tororo, Manafwa, Butaleja and Busia) and, whenever outbreaks occur, throughout the country.

Such data may be combined with the available national reporting statistics to better model cholera burden within Uganda, which in turn may be used to conduct economic analyses (e.g., cost effectiveness or cost benefit studies) of the potential use of cholera vaccines in Uganda. Given the health challenges facing Uganda, the decision to pursue cholera vaccination must be weighed against the introduction of other health interventions that may have a greater impact on mortality (e.g., pneumococcal conjugate vaccines, rotavirus vaccines, future malaria vaccines or other interventions).

In addition to targeting high-risk endemic populations, Uganda may consider using cholera vaccines from a recently established international stockpile to mitigate epidemic cholera. A review of 15 epidemic curves showed that about 57% of the cases occurred after six weeks across all outbreaks. This 57% figure may represent an upper bound on the number of cases that could be averted via reactive use of cholera vaccines from a global stockpile, assuming it would take at least three weeks to diagnose an outbreak and prepare for a mass vaccination campaign plus three weeks to generate immunity from the two-dose vaccine. This stockpile may also be used to prevent the spread of cholera to neighboring districts, such as when it was used in an Adjumani district refugee camp in 1997 [18].

While cholera incidence in Uganda has been manageable over the past decade, elimination of the disease is likely to take time especially given the slow progress on provision of safe water and sanitation among other risk factors. Most of the areas with the highest incidence rates either border countries with political instability and endemic cholera (e.g., DRC and Sudan) or contain semi-nomadic populations. For these districts, it would be difficult to prevent cholera-infected persons from crossing borders, achieve high vaccination coverage rates, or to construct reliable water and sanitation infrastructure for semi-nomadic populations [19].

Some global trends in cholera disease burden may lead to an increase in the number of cases and should be considered in cholera control planning. At present, cholera is more prevalent in rural areas than in urban areas within Uganda. This may change if present urbanization trends continue and the maintenance and expansion of water and sanitation infrastructure cannot keep pace with the rapidly growing urban population. The urban population in Uganda is projected to increase more than seven-fold from 4.5 million in 2010 to 31 million by 2050 [20].

Some studies have found multidrug resistant *V. cholerae* in Uganda, including strains resistant to trimethoprim, sulfonamides, ampicillin, tetracycline, chloramphenical and streptomycin [21]. In addition, there is evidence that the severity of clinical cases of cholera in Asia and Africa is increasing, especially during outbreaks. Some scientists attribute the increase in severity of cholera cases seeking treatment to the emergence of a new altered strain of *V. cholerae* O1 El Tor that secretes the classical cholera toxin, making it more virulent [22]. It is not presently known if this strain is present in Uganda. However, it has been isolated from recent African outbreaks in Mozambique and Zimbabwe [23], [24].

Due to global warming, the average temperature in Uganda is estimated to increase by up to 1.5 degrees over the next 20 years [25]. Recent research suggests a strong correlation between increased rainfall and elevated temperatures with higher cholera incidence [26], [27], [28], [29], [30]. This may pose an elevated risk for districts bordering Lake Albert and Lake Victoria, which may provide an endemic reservoir of *V. cholerae* [31], [32].

There are also trends suggesting a reduced need for cholera vaccination in Uganda. The multidisciplinary cholera outbreak response activities have been effective in mitigating the severity of outbreaks, both in terms of morbidity and mortality. The cholera case fatality rate has steadily declined since the large, nationwide outbreak in 1998 (refer to *Figure 1*). While improving treatment does not reduce the incidence of cholera cases, it does reduce the social and economic burden of the disease.

Improvements in access to improved water and sanitation would also lead to a concomitant decrease in cholera incidence. These cholera incidence data may also be used to target priority districts for improvements in water, sanitation, and hygiene efforts. Cholera incidence is likely to be associated with high prevalence of other enteric diseases, for which cholera vaccination would have no effect. Considering that an estimated 30,000 persons die from diarrheal disease every year in Uganda, improved water, sanitation, and hygiene are urgently needed even if cholera vaccine is deployed.

In conclusion, the existing surveillance system is geared toward mitigating the impacts of cholera outbreaks, not quantifying the burden of endemic cholera. Cholera control activities have been effective in slowing the spread of cholera and reducing cholera fatalities. However, cholera cases continue to be reported on an annual basis. The combination of sentinel surveillance with national cholera incidence data could be used to develop an economic analysis to inform cholera vaccination policy.
